# The situational analysis of teaching-learning in clinical education in Iran: a postmodern grounded theory study

**DOI:** 10.1186/s12909-022-03577-3

**Published:** 2022-07-02

**Authors:** Soleiman Ahmady, Hamed Khani

**Affiliations:** Department of Medical Education, Virtual School of Medical Education & Management, Shahid Beheshti University of Medical Sciences, Valiasr St., Parkway, Tehran, Iran

**Keywords:** Research in education, Teaching & learning, Undergraduate medical education, Educational development, Grounded theory, Situational analysis

## Abstract

**Background:**

Clinical teaching-learning is a context-bound phenomenon. One of the problems related to field of medical education research is the lack of sufficient attention to context-appropriate methodologies. The purpose of this qualitative inquiry is to explain and represent teaching-learning in the clinical education of general medicine in Iran using the three types of maps situational, social worlds/arenas, positional, in combination with discourse analysis.

**Methods:**

In this study, the authors used the situational analysis approach as a postmodern version of grounded theory. The data collection was undertaken in three stages. In the first stage, a mini literature review was conducted to highlight a possible gap in applying situational analysis in medical education research and the development of this methodology. In the second stage, the latest and most up-to-date documents of the Ministry of Health and Medical Education (MOHME) of Iran, the general medicine curriculum, and related documents were analyzed. Finally, the remote semi-structured interviews (web-based and telephone) were undertaken in the third stage. Participants in this stage included expert clinical teachers, medical education specialists, and students. In this study, the notes and transcripts were analyzed for the emergence and categorization of sub-themes and themes, represented in three maps.

**Results:**

Thirty-one participants were involved in the web-based interviews, while seven participants took part in the telephone interview. Based on this research, the teaching-learning situation in clinical education on general medicine in Iran was represented in three maps; situational, social worlds/arenas, and positional. In addition, the results showed, clinical education of general medicine in Iran in six positions (curriculum; culture, behavior and attitude; management and leadership; environment, space and time; financial; and technology) has serious problems and challenges. Finally, based on the horizontal axis of the positional map, recommendations were provided to develop and support effective clinical teaching.

**Conclusions:**

The clinical learning environment is a complex and multi-layered social environment in which should be considered these numerous social layers, arenas, social worlds, and discourses while developing curricula and teaching.

**Supplementary Information:**

The online version contains supplementary material available at 10.1186/s12909-022-03577-3.

## Background

Clinical education plays an important role in medical education since it provides the opportunity for medical students to learn. The clinical practice is an essential and irreplaceable component and resource in preparing medical students for their professional roles (professional identity) [1]. Clinical education can be considered a set of activities that facilitate learning in the clinical environment, which is to make measurable changes in the students’ knowledge, attitude and skills to perform clinical care. Clinical education provides medical students with an opportunity to develop competency and skillsets to function within dynamic and complex settings. The importance of clinical education has been considered throughout the literature as a vital and essential element for the continuous growth of students [2].

Teaching-Learning in the clinical environment is the heart of medical education and various factors affect it [3–6]. Medical students acquire clinical knowledge, skills, and attitudes in clinical settings and think, feel, and act as a physician [3, 7]. In fact, in this environments, students learn what a real doctor means.

Teaching-Learning in a clinical setting focuses on patients and their problems. In these environments, students learn skills such as; history taking, physical examination, patient communication, and professionalism in the best way, and they apply medical knowledge directly to patient care [8, 9].

Research in clinical medical education requires attention to appropriate and context-sensitive methodologies. The situational analysis approach is one of these methods.

The situational analysis method is not mainly well developed in medical education research. The research body of this method is weak in medical education research. Various research has been done about the analysis of the situation of clinical education, but not with the method of situational analysis as one of the branches of grounded theory.

For example, Myers & Covington [10] in a study analyzed the situation of clinical education in general medicine. In this study, the authors focused on identifying the root causes of student failure in the United States and their failure to achieve educational objectives and outcomes. Using principal cause analysis processes, they designed and implemented a framework that could be used to identify the causes of students’ failure in clinical education.

Kiguli et al., [11] in a study focused on the situational analysis of teaching and learning of medicine and nursing students at Makerere University College of Health Sciences. The data of this study were collected through documents and curriculum analysis, interviews, and focus groups. The core competencies that medicine and nursing students are expected to accomplish at the end of their training have been described based on this research for both programs. The curricula are in the process of reform toward competency-based education and seemingly aligned well with Uganda’s strategic needs. However, implementation is insufficient and should be improved so that learning objectives support skill development. Learning experiences are more relevant to competencies and future situations. The competency evaluation process is better planned.

Clinical teaching-learning is a phenomenon that is impacted by the context. The student in this learning environment is immersed in the clinical environment’s culture and acquiring knowledge, skills, and problem-solving strategies [12]. Therefore, representing and explaining this special situation using the situational analysis approach is of particular importance.

In addition, according to studies, developing and improving the quality of teaching-learning in clinical education requires explaining the current situation and identifying the challenges and problems and its strengths.

Heydari, DadgarMoghaddam, and Ebrahimi Garoui [13], in a qualitative study, addressed the challenges of general medical education in the school of Medicine, Mashhad University of Medical Sciences. The data analysis extracted four main categories or themes: educational structure (curriculum), teaching and learning activities, professional interaction, and resources.

Changiz, Yamani & Shaterjalali [14], in a triangulation study, examined the challenges of planning learning opportunities for clinical medicine in Iran. Student-centered learning, non-threatening learning settings, and management of clinical learning opportunities were identified as implementable learning opportunities. According to the findings of this research, these items in medical schools were not well established. Also, the results of this study showed that determining content-based learning opportunities and ongoing supervision of learners to achieve expected learning outcomes were among the clinical learning opportunities with more than 70% consensus on them. According to the results of these studies, effective teaching in clinical education can be achieved by removing these challenges.

In general, while paying attention to methodological development in this research, we can pave the way for educational development by focusing on the unique features of the situational analysis method. In this regard, the specific research questions were as follows: 1) what are the fundamental components and elements (human, non-human, material, symbolic and discursive) of teaching-learning in clinical education of general medicine in Iran? 2) What are the arenas of clinical education in general medicine in Iran? 3) What are the worlds and social discourses in arenas of clinical education? 4) What are the challenges of clinical education in general medicine in Iran?

## Aims of this study

This study aimed to explain the fundamental components and elements of teaching-learning and identify the challenges and problems that stand in the way of effective teaching-learning in clinical education of general medicine in Iran.

## Methods

### Research approach

This qualitative study was conducted using the situational analysis approach. The context is essential in medical education; we chose the situational analysis method to explore clinical education because this method targets context. Clarke [15], developed situational analysis based on Anselm Strauss’s legacy. Although grounded theory and situational analysis are fundamentally rooted in social constructionism and seek to explore the diversity of perspectives and the processual and contingency nature of social life by a relational ecological framework, Clarke [15], has critiqued grounded theory and developed situational analysis that is situation-centered (primarily context-driven). She believes it addresses shortcomings in traditional ground theory such as positivist tendencies, a lack of reflexivity, simplicity rather than addressing differences, and a lack of power analysis. One of the key advantages of situational analysis is that it provides a reliable and rigorous method for resolving complicated issues in research [16]. Also, situational analysis is rooted in symbolic interactionism and pragmatist philosophy. This approach also has new roots, such as analyses of Foucault’s discourse that go beyond “the knowing subject,” paying special attention to and explicitly acknowledging non-human actors and analyzing implicated actors and actants. The juxtaposition of these cases has led to the transition to the situation as the center of gravity of the analysis and its perception as an analytical unit [16].

### Data collection and triangulation

The data of this study were collected using a combination of multiple methods and sources. In other words, the data were collected through the methods of literature review, analysis of the general medicine curriculum and related documents, remote qualitative interviews (web-based and telephone). The data of this study were collected from July 10 to August 16, 2021.

#### Mini literature review

This step was conducted to be aware of the use of the situational analysis method as a post-structural version of grounded theory in the research practice of medical education. In other words, the purpose of this mini literature review was to highlight a possible gap in the application of situational analysis in medical education research and the development of this methodology. In this regard, the researchers sought to answer this question: What is the current status of using situational analysis in medical education research?

Persian and English articles were searched in three databases, PubMed, Web of Science, and Google Scholar, from 2010 to 2021. Keywords of “Research”, “Medical education”, “Qualitative methods”, “Clinical education” or “Clinical teaching”, “Situation”, “Methodology”, “Situational analysis”, “Grounded theory”, “Reflexive methodology”, “Clarke’s approach”, “Methodological development” and terms related to each of these keywords were searched. After removing duplicates and screening the titles and abstracts, the ten most relevant articles on situational analysis were identified, and none of them used Clarke’s situational analysis method was not performed.

#### Documents and curriculum analysis

In this step, the documents of the Ministry of Health and Medical Education (MOHME) of Iran, such as; the Curriculum of Undergraduate Medical Education (General Medicine curriculum) in Iran [17], national standards of general medicine [18], fundamental standards of Undergraduate Medical education In medical schools and higher education centers of Iran [19], Document of expected competencies of general medicine graduates [20], clinical education standards [21], clinical education criteria of general medicine program [22], Executive Regulations for dress code and students’ professional ethics in the laboratory and clinical environments [23], and charter of patients’ rights in Iran [24] were analyzed. This step was conducted to identify the elements and components (human, non-human, material, symbolic and discursive) and the arenas of teaching-learning in clinical education of general medicine. In addition, the analyzed content of these documents was used for the development of remote qualitative interviews questions.

#### Semi-structured interviews (web-based and telephone)

The web-based interviews were used to acquire a broad understanding of the situation, followed by the telephone interviews (video and voice call) to gain a rich and deep insight into the people involved. At this stage, the data collection process was designed to identify the elements and aspects (human, non-human, material, symbolic and discursive), including the importance of each of these elements and components in the teaching-learning situation, discovering the arenas of teaching-learning and the social worlds present in them, identifying the components and elements that facilitate learning and achieving learning goals and outcomes, and identifying the challenges of clinical education in relation to teaching-learning from the perspective of those engaged in the situation. Interviews were conducted with expert clinical teachers, medical education specialists and students.

The questions were uploaded to Pressline, and participants were provided a web-based interview link via WhatsApp instant messenger after they were prepared. We invited forty people for web-based interviews. None of the web-based interview respondents participated in the telephone interview. Web-based and telephone interviews questions were the same, with the difference that in the telephone interviews additional questions were developed during the conversations. However, many medical educators, clinical teachers, and students received web-based interview questions via Porsline and answered only the questions included.

All interviews were conducted by the second researcher who is qualified in conducting qualitative interviews. The data collection through interviews continued until saturation and theoretical adequacy. Five participants agreed to have their voices recorded during the telephone interviews, and the second researcher transcribed their recordings verbatim. The other two refused to have their voices recorded, so the content of their interviews was noted and transcribed during the interview. An introductory question was asked to establish rapport with the interviewees, followed by five key and main open-ended questions. The five recorded interviews lasted 30 minutes on average (between 15 and 40 minutes), while the two immediately noted and transcribed interviews lasted 45 minutes on average (between 30 and 60 minutes).

### Study context

Undergraduate Medical Education (UME) in Iran has two courses that include four stages, pre-clinical course (including two stages of basic sciences and physiopathology/semiology) and clinical training (including two stages of clinical clerkship and internship). The focus of this inquiry is the clinical settings and teaching-learning in clinical training.

### Study participants

There are two groups of participants in this study; the first category of humans includes; expert clinical teachers, medical education specialists and students, and the second category of non-human beings such as; analysis of the general medicine curriculum and related documents. Table 1 shows the human participants and the method of their selection.

**Table 1:** Participants and their selection methods

Sampling method	Convenience sampling	Maximum variation sampling	Snowball sampling
Participants			
Analysis of the general medicine curriculum and related documents	**✓**		
People from different groups including; expert clinical teachers, medical education specialists and students (web-based interview)		**✓**	
Telephone interview with specialists and key informants (expert clinical teachers and medical education specialists)			✓

We recruited fifth, sixth, and seventh-year students who were practically engaged in the clinical education practice; they were 23 to 27 years old. The students were from Tehran and Shahid Beheshti Universities of Medical Sciences. The expert clinical teachers and medical education specialists were from Tehran, Shahid Beheshti, Shiraz, Guilan, and Ahvaz Jondishapur universities of medical sciences, whose ages ranged from 40 to 56 years.

The criteria for the expertise of medical education specialists included having a Ph.D. in medical education from Iran or abroad, being a faculty member with the academic rank of (Assistant professor, Associate professor, and professor) and at least ten years of work experience in the field and practice of medical education (educational activity, teaching, and Scholarship of Teaching and Learning/ SOTL).

The expert clinical teachers were specialists in pediatrics, internal medicine, social medicine, and family medicine. The expertise criteria included having expertise in one of the clinical disciplines, faculty member, at least fifteen years of work experience in a specialized field, educational activities, teaching and academic rank (Associate professor and professor).

Based on the reputational case, the first person and then other specialists, one after the other, entered the study process in a snowball sampling.

### Data analysis (mapmaking as an analysis tool)

In this study, the notes of the documents and curriculum analysis and the transcripts of the qualitative interviews (web-based and telephone) were analyzed for the emergence and categorization of sub-themes and themes. The themes that emerged from each source’s data (documents and curriculum analysis, and remote qualitative interviews) were classified independently, then overlapping and comparable sub-topics and themes were deleted by comparing them. Finally, the themes from all the data sources were aggregated to achieve a holistic view of the situation and represented in three maps. The main strategies of situational analysis are the three maps that researchers perform across the full path of the research project process (from design to report). The first map is a situational map, which analyzes the research situation at a macro level. These maps enable the researchers to articulate the major elements in the situation from their own ontological perspectives. The second map, the social worlds/arenas map, depicts all of the collective actors as well as the arena(s) of commitment in which they are involved in ongoing discourses and negotiations. This map is a meso-level analysis. The third map is the positional map. This map is a micro-level analysis and focuses on the main positions taken and not taken in the data. In general, situational analysis can deeply study research projects individually, collectively, organizationally, temporally, geographically, materially, discursively, culturally, institutionally, visually, symbolically, and historically.

### Memos and memoing

Memoing is an analytical process that ensures the quality of the grounded theory, particularly situational analysis [25]. According to Stern [26], if data are the building blocks of a developing theory, memos are the mortar. In this study, the researchers employed theoretical, methodological or operational and diagrammatic memos to represent the evolutionary analysis of the situation, particularly relational analysis on situational maps, during and immediately after gathering data from qualitative interviews (web-based and telephone). Examples of these are presented in the Supplementary information file (See Supplemental 1, 2, and 3).

### Rigor and trustworthiness

A qualitative inquiry’s rigor and trustworthiness are critical components. In fact, if a reader of a study report can audit the data collecting and analysis procedure, the study is likely to be reliable [27]. Lincoln and Guba’s criteria were used to increase the trustworthiness of the data in this study [28]. Table 2 presents these criteria and the strategies that guarantee them.

**Table 2:** Criteria for the trustworthiness of data and them guaranteeing strategies

Criteria	Credibility	Dependability	Confirmability	Transferability
Strategies				
Use of memos and memoing	✓	✓		
Prolonged engagement with data	✓	✓		
Member checking	✓	✓		
Peer checking	✓	✓		
Coding and categorizing of the emerged themes by the researcher, thesis supervisor and a qualitative research expert and reaching a consensus	✓	✓		
Allocating sufficient time to data collection and analysis			✓	
Using the utmost precision in the research process			✓	
Use of audit trail or documentation all stages and procedures of research			✓	
Validation and quality assessment of findings by two medical education specialists and expert clinical teacher				✓
Use of different participants in terms of position				✓

As shown in Table 2, we used different strategies to ensure data rigor and trustworthiness. One of these strategies was memoing. The second researcher used it during the data collection process and transcribing qualitative interviews responses to find out the depth of the data, strong interaction with the data, and sensitivity to the meaning of the data. Another of these strategies was prolonged engagement with data. Given that the present study is one of the sub-studies of the Ph.D dissertation, the researchers were in contact with the setting and the research participants for one year. In addition, when collecting data related to this article, the second researcher was in constant contact with its participants via WhatsApp and telephone. Member checking was another strategy in which the authors referred to participants, especially telephone interviewees, to confirm emerging themes and study findings. Another strategy was peer checking, for which we consulted with two peer students working in the field of qualitative inquiry. In addition, the findings of the dissertation and the findings of this section were presented in two sessions of pre-defense and defense of the dissertation in the presence of the supervisor, internal and external reviewers and peer students.^1^ Another strategy for ensuring data trustworthiness was coding and categorizing themes from all the data sources (curriculum analysis and related documents, and remote qualitative interviews) performed jointly by the main researcher, supervisor, and peer students. During the present research process, sufficient time was allocated to data collection and analysis to ensure the trustworthiness of the data, maintain the accuracy of the research process, prolonged engagement with data, member checking, peer checking, and triangulation methods were used. Another strategy was the audit trail, which used the agreement of two researchers (two peer students) to follow the authors’ decisions. The views of these two peer students indicated that the audit or decision-making cycle of all decisions made by researchers could be traced. The findings of this study were validated by two medical education specialists and clinical teacher who were not among the study participants and the quality of the findings was evaluated. Another strategy to ensure data rigor was using different participants (expert clinical teachers, medical education specialists and students).

Finally, to write this manuscript, practical steps related to guarantee five key criteria (plausibility, relevancy, consistency, transparency and currency) for the quality of qualitative research were considered [29]. These practical steps serve as a checklist and authors and researchers can use them in preparing qualitative inquiry manuscripts and reports.

## Results

### Description of participants

Thirty-one people responded to the web-based interview questions out of the invited forty. Nine were medical education specialists and clinical teachers, and twenty-two were fifth, sixth, and seventh-year medical students (interns).

In Table 3 the number of participants in the semi-structured qualitative interviews (web-based and telephone) for the groups of clinical teachers, medical education specialists, and students is reported separately.

**Table 3:** Number of human participants in the research

Descriptive statistics	N	The sum of each group	Percentage
Participants			
Expert clinical teachers in web-based interviews	2	6	15/8
Expert clinical teachers in telephone interviews	4		
Medical education specialists in web-based interviews	7	10	26/3
Medical education specialists in telephone interviews	3		
Students in web-based interviews	22	22	57/9
Total	38		100/0

The web-based interviews included twenty-two students, fourteen of whom were male and eight female. The average age of the participants was (24.4) years. Also, five men and four women were among the nine medical education specialists and clinical teachers.

Seven medical education specialists and clinical teachers were also telephone interviewed. Of these seven specialists, four people were male and three people were female. The mean age of specialists and clinical teachers was (48.6) years.

### Mapmaking and presenting maps

The situational analysis employs a cartographic representation of the study topic, with three maps depicting research analyses at various levels. These maps are important analytical tools that can act as discursive tools for creating communities or collections, provide a visual representation of complexity, visualize the research questions, and generate new and different insights.

This section analyzed data from various sources (curriculum analysis and related documents, and remote qualitative interviews) in light of the research questions. The resulting themes were represented in situational analysis maps, which are shown below.

#### Presenting situational maps

The fundamental question to be answered in creating the situational maps is: Who and what are in the broader situation?

In this research, the question is: What are the components and elements (human, non-human, material, symbolic and discursive) of the teaching-learning situation in the clinical education of general medicine in Iran?

To answer this question, situational maps were represented in two versions: messy version and ordered version.

##### Presenting situational map: messy version

**Fig. 1:**
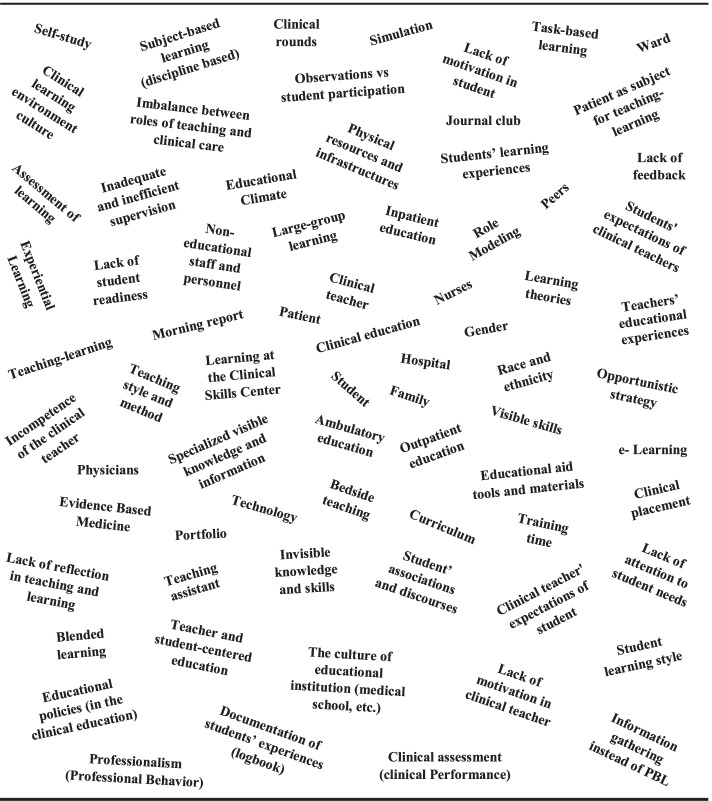
Messy situational map: components and elements of the teaching-learning situation in clinical education of general medicine in Iran

According to this map, a situation encompasses all human, nonhuman, material, symbolic, and discursive components and elements and is constructed and represented by the people involved in the situation and the researchers. Figure 1 represents the components and elements of the teaching-learning situation in clinical education of general medicine in Iran.

To represent the messy map of the situation, the second researcher asked participants to write down all the components and elements in the situation under study. Also, the researcher wrote down all the components and elements in this situation based on documents. This is precisely what the situational map should be; thus the researcher did not limit himself to providing an organized list and instead invited the participants to think freely, extensively, and with a big picture in mind about the topic under investigation. They were asked to present all the components and elements they have experienced about the situation, no matter how marginal they may seem. This step of situational analysis allowed the researcher to tell stories without removing important aspects of the situation.

##### Presenting situational map: ordered version

The discourses and themes that emerged in the messy version can be organized and specifically represented using the ordered version of the situational map. Table 4 presents the discourses and themes that emerged in the previous step in an ordered and categorized manner.

**Table 4:** Ordered situational map: components and elements of the teaching-learning situation in clinical education of general medicine in Iran

**Individual human elements/ actors** (such as; key individuals and important [unorganized] people in situation, etc.)	**Non-human elements actors/ actants** (such as; technologies, material infrastructures, specialized information and/ or knowledges, material things, etc.)
• Clinical teacher • Student • Patient • Physicians • Nurses • Non-educational staff and personnel • *Teaching assistant* • peers	• Technology • Physical resources and infrastructures • Specialized visible knowledge and information • Assessment of learning • Simulation • Clinical assessment (clinical Performance) • Learning theories • Curriculum • *Portfolio* • Educational aid tools and materials • Documentation of students’ experiences (logbook) • Learning at the Clinical Skills Center • Invisible knowledge and skills • Visible skills • Information gathering instead of PBL • Students’ learning experiences • Teachers’ educational experiences
**Collective human elements/ actors** (such as; particular groups, specific organizations, etc.)	**Implicated/ silent actors/ actants** (as found in the situation)
• Hospital • Student’ associations and discourses • Large-group learning • Family • Morning report • *Journal club* • Clinical rounds	• Patient • Family
**Discursive constructions of individual and/ or collective human actors** (as found in the situation)	**Discursive constructions of non-human actants** (as found in the situation)
• Patient (only) as subject for teaching-learning • Incompetence of the clinical teacher • Lack of attention to student needs • Lack of motivation in student • Observations vs student participation • Lack of student readiness • Lack of motivation in clinical teacher • Lack of reflection in teaching and learning • Blended learning	• Technology • Simulation • *Evidence Based Medicine* • Subject-based learning (discipline based) • Task-based learning • Learning at the Clinical Skills Center
**Political/ economic elements** (such as; the state, local/ regional/ global orders and institutions, political parties, non-governmental organizations [NGOs], politicized issues, etc.)	**Sociocultural/ symbolic elements** (such as; religion, race, sexuality, gender, ethnicity, nationality, logos, icons, other visual and /or aural symbols, etc.)
• Educational policies (in the clinical education) • Expand services and ambulatory and outpatient educational environments • Blended learning	• Gender • Race and ethnicity • Clinical learning environment culture • The culture of educational institution (medical school, etc.) • *Role Modeling* • Professionalism (Professional Behavior) • Visible skills
**Temporal elements** (such as; historical, seasonal, crisis, and/or trajectory aspects, etc.)	**Spatial elements** (such as; spaces in the situation, geographical aspects, local, regional, national, global spatial issues, etc.)
• Training time • Clinical education • Assessment of learning • Lack of feedback • Invisible knowledge and skills • Clinical placement • Imbalance between roles of teaching and clinical care • Students’ learning experiences • Clinical teachers’ educational experiences	• Ward • Inpatient educational environment • ambulatory educational environment • outpatient educational environment • Bedside teaching • Educational *Climate* • Teaching-learning situation • Clinical education • Invisible knowledge and skills • Clinical Skills Learning Center (CSLC) • Clinical placement • Students’ learning experiences • Clinical teachers’ educational experiences • Experiential learning (workplace learning/ reflection in action) • Inadequate and inefficient supervision • Lack of feedback
**Major issues/ debates [usually contested]** (as found in the situation)	**Related discourses (historical, narrative, and/or visual)** (such as; normative expectations of actors, actants, and/or other specified elements; mass media and other popular cultural discourses; situation-specific discourses, etc.)
• Applying e-learning and online in clinical education • Opportunistic strategy (lack of a systematic program in clinical education) • Imbalance between roles of teaching and clinical care • Information gathering instead of PBL • Patient (only) as subject for teaching-learning	• Learning theories • Subject-based learning (discipline based) • Task-based learning • Experiential learning (workplace learning/ reflection in action) • Students’ expectations of clinical teachers • Clinical teacher’ expectations of student • Teacher and student-centered education (simultaneously)
**Other kinds of elements** (as found in the situation)	
• Teaching style and method of clinical teacher • Student learning style • Self-study	

Table 4 shows the components and elements of the teaching-learning situation in clinical education of general medicine in Iran using the ordered version of the situational map in different categories. It should be emphasized that, despite their fixed appearance, these maps are not static and can include a great deal of dynamism and fluidity. When categorized into an ordered map, however, each of the components, elements, discourses, and themes that emerged in the Messy form of the situational map can exist in more than one category, as shown in Table 4. In general, the representation of these components and elements in the ordered version is important to reveal the complex conditions in the situation (here, teaching-learning in clinical education of general medicine in Iran).

### Presenting relational analysis with situational maps

After creating messy and orderly versions of the situational map, the next step is to pose the question based on these maps and the researcher’s memos. In fact, in this step, the researcher pondered the situational map and represented important relationships. These relational maps assist the researchers in deciding which stories and relations to explore as an analyst. In the present study, relational analysis was represented for clinical education (Supplemental 4) as well as teaching-learning in clinical education (Supplemental 5). (See Supplementary information file).

#### Presenting social worlds/arenas map

**Fig. 2:**
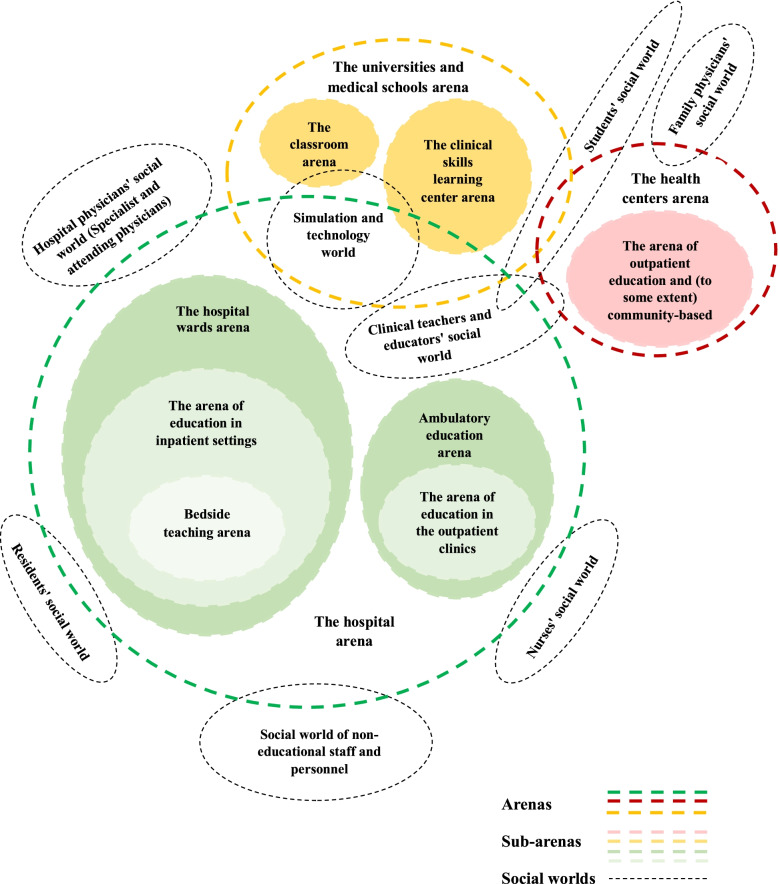
Social worlds/arenas map: social worlds in arenas of teaching-learning in clinical education of general medicine in Iran

Figure 2 represents the social worlds in arenas of teaching-learning in clinical education of general medicine in Iran.

Teaching-learning in clinical education of general medicine in Iran has arenas and sub-arenas. In fact, arenas such as hospitals, health centers (comprehensive health services), and universities and medical schools can be represented for clinical training in general medicine (see Fig. 2). One of the arenas of teaching-learning in clinical education of general medicine in Iran is the hospital, which includes sub-arenas such as; hospital wards (education in inpatient settings and bedside teaching) and ambulatory education (education in the outpatient clinics), the clinical skills learning center. Another arena of teaching-learning in the clinical education of general medicine in Iran is the health centers/ comprehensive health services (which include outpatient education and [to some extent] community-based education). Finally, other arenas of teaching-learning in clinical education of general medicine in Iran are the universities and medical schools (which include the clinical skills learning center and classrooms).

In addition, as shown in schematic Fig. 2, different social worlds are identified in the arenas of teaching-learning of clinical education. In the hospital arena and its sub-arenas, there are social worlds such as the clinical teachers and educators’ social world, students’ social world, hospital physicians’ social world (specialist and attending physicians), residents’ social world, nurses’ social world, the social world of non-educational staff and personnel and simulation and technology world. In the health centers arena (comprehensive health services) and its sub-arenas, there are social worlds such as the clinical teachers and educators’ social world, students’ social world and family physicians’ social world. Finally, there are social worlds in the universities and medical school arenas and their sub-arenas, such as the clinical teachers and educators’ social world, students’ social world, and simulation and technology world.

#### Presenting positional map

**Fig. 3:**
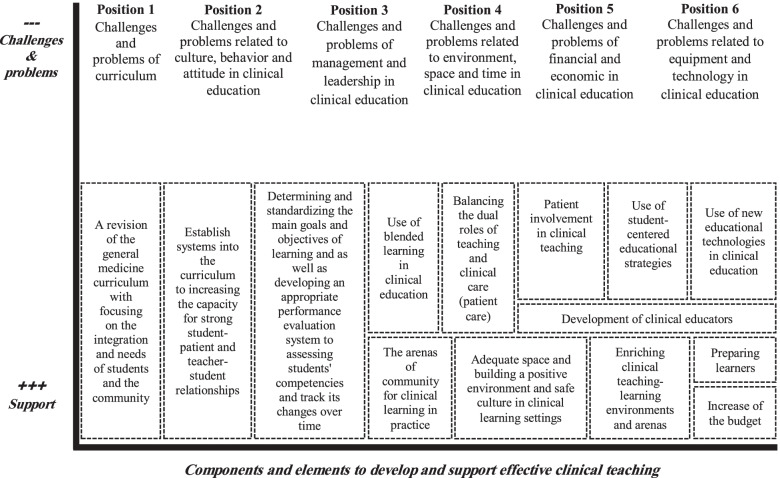
Positional map: Positions of clinical education of general medicine in Iran with a pathological view

The positional map is the final category of map used to chart data. It shows various positions within major discursive issues arising in the data (Fig. 3).

The positional map illustrates positions of clinical training of general medicine in Iran with a pathological view (see Fig. 3). The vertical axis shows the challenges and problems related to the clinical education of general medicine in Iran in 6 positions, including challenges and problems of curriculum (position 1), challenges and problems related to culture, behavior and attitude in clinical education (position 2), challenges and problems of management and leadership in clinical education (position 3), challenges and problems related to the environment, space and time in clinical education (position 4), challenges and problems of financial and economical in clinical education (position 5) and challenges and problems related to equipment and technology in clinical education (position 6). The horizontal axis also shows the components and elements for developing the effective clinical teaching. Finally, both the vertical and horizontal axes help in the organization of the items on the map, allowing the range of possible places to be explored.

In Supplemental 6 the challenges and problems obstacles to effective teaching-learning in clinical education are presented based on six positions (See Supplementary information file).

## Discussion

### Key findings and their relation to the existing literature

While there is research on the analysis of the situation of clinical education in general medicine [10], the challenge of planning learning opportunities for clinical medicine [14], barriers of teaching and learning in clinical rounds [30], challenges of clinical education in undergraduate medical education [13], and situational analysis of teaching and learning of medical students through curriculum analysis and focus group interviews [11]. There has been no research done using situational analysis (SA) as an extended form of the grounded theory (GT) method in this regard. Because of the use of Clarke’s situational analysis method, which is a post-structural discourse that focuses on context, the current study is unique in medical education research.

The teaching-learning system in general medicine clinical education, with an emphasis on its challenges, was represented in three maps in this study using the situational analysis method. All human, nonhuman, material, symbolic, and discursive elements were shown from the perspective of the people involved in the situation using situational maps, and then all of these elements and aspects were categorized using the ordered version.

In addition, according to results of this study, when it comes to situational maps, it can be noted that because the data for this study was collected from a variety of sources and methods, there were some interesting findings in terms of the components and themes that emerged. Human, non-human, symbolic, and discursive components and elements such as community-based medical education, small groups learning, problem-based learning, attention and focus on learners’ needs, systematic approach in clinical education, and others have been considered in the clinical training of general medicine curriculum, while the results of the semi-structured qualitative interviews (web-based and telephone) do not indicate this. As a result, there is no alignment or conformance between the planned and intended curriculum, what teachers teach (the delivered curriculum), and what the students learn (the experienced curriculum). In fact, according to research by Kiguli et al. [11], sometimes, what is expected of a curriculum is not reflected in practice.

The data of this study showed that teaching-learning situations in clinical education of general medicine in Iran has arenas such as hospitals, universities and medical schools and health centers (comprehensive health services). The hospital arena includes sub-arenas such as hospital wards (education in inpatient settings and bedside teaching), ambulatory education (education in outpatient clinics), and the clinical skills learning center. Other arenas of teaching-learning in clinical education of general medicine in Iran are the universities and medical schools (which include the clinical skills learning center and classrooms). Health centers (comprehensive health services) are almost like other arenas of teaching-learning, which include outpatient education and [to some extent] community-based education. Although the findings of previous research shows that clinical education has shifted from bedside teaching to classrooms and corridors [31], clinical education in general medicine in Iran remains primarily hospital-based, despite the findings of this study and the identification of three main arenas.

Another finding based on multiple data sources and methods is that the community-based education arena has been considered as one of the arenas of clinical education in general medicine in the documents and curriculum. Still, the data collected from the semi-structured qualitative interviews do not support this. Furthermore, many expert clinical teachers, medical education specialists, and students believe that health centers were originally designed for this purpose. In interviews, several professional clinical teachers and medical education specialists stated that the COVID-19 pandemic gave an outstanding and remarkable chance for general medicine clinical training that would be continuous from hospital to community-based education.

Another finding based on multiple data sources and techniques is that there is a gap between expert clinical teachers and medical education specialists in Iran regarding teaching-learning arenas in general medicine clinical education. According to several medical education specialists. Clinical teachers who teach believe that, despite these arenas being the curriculum’s focus, clinical education in general medicine in Iran is primarily hospital-based (inpatient, outpatient and based on clinical skills learning center). This paradox, in general, conveys the notion of the divide between medical education professionals and clinical teachers or the gap between theory and practice.

Furthermore, the social worlds in the arenas of teaching-learning in the clinical education of general medicine in Iran were represented on the second map. In the hospital arena and its sub-arenas, there are social worlds such as the clinical teachers and educators’ social world, students’ social world, hospital physicians’ social world (specialist and attending physicians), residents’ social world, nurses’ social world, the social world of non-educational staff and personnel and simulation and technology world. There are social worlds in the health centers arena and its sub-arenas, such as the clinical teachers and educators’ social world, students’ social world, and family physicians’ social world. Finally, there are social worlds in the universities and medical school arenas and their sub-arenas, such as the clinical teachers and educators’ social world, students’ social world, and simulation and technology world.

In reality, in the teaching-learning situation in general medicine clinical education in Iran, some persons and groups have more connection and become closer to each other’s social worlds, such as clinical teachers and educators’ social worlds and students’ social worlds. Also, some of these worlds participate in more than one arena, such as the clinical teachers and educators’ social world and students’ social world participating in all three arenas of hospitals, universities, medical schools, and health centers. The important point about the social worlds/arenas map is that no patients’ social world is represented in the arenas for clinical education. In general medicine clinical education in Iran, patients are only treated as “subjects for teaching-learning.” They are not allowed to participate in clinical teaching, curriculum design, or evaluation. In other words, they are present in these arenas, but not as collective actors in education, but as implicated/ silent actors/ actants, as explained by Foucault’s discourse, which indicates that the patient’s voice is not heard in relation to educational involvement. According to Foucault’s book “The Birth of the Clinic” [32] patients are only examined medically in the new era. Therefore, it demands that clinical education be humanistic in both education and service delivery and care.

Another important factor to consider is that, in the hospital arena, providing clinical care takes precedence over teaching because of the presence of hospital physicians’ discursive and social worlds (specialist and attending physicians), nurses’ discursive and social worlds, residents’ discursive and social worlds, and the social world and discursive of non-educational staff and personnel. This important finding can also be explained by Foucault’s discourse; it may be stated that the power of specialists and attendings, residents, and nurses in the hospital arena has dominated education in this context. Thus, this has led to the imbalance between the dual roles of education and clinical care in the clinical education system of general medicine.

Finally, the final map was plotted challenges and problems related to clinical education that are obstacles to effective teaching-learning (vertical axis) and components and elements to construct and support effective clinical teaching (horizontal axis), both based on research data.). The challenges and problems that obstruct successful teaching-learning were conceptualized and categorized according to this Biaxial diagram in six positions as (curriculum/ culture, behavior and attitude in clinical education/ management and leadership in clinical education/ environment, space and time in clinical education/ financial and economical in clinical education and/ equipment and technology in clinical education).

### Study strengths and limitations

Our research has several advantages. Based on our search in the medical education literature, this study is the first to use postmodern grounded theory (situational analysis). Also, from the methodological point of view, this study promotes the use of the situational analysis approach to study complex systems. It encourages medical education researchers to continue to improve methodological development in medical education research. The methodology of situational analysis is mainly situation-centered (context-driven). This type of study can develop a relationship between context (practice) and research because of the relevance of context in medical education. This was another advantage of this study. In addition, multiple groups of participants, including clinical teachers, medical education professionals, and students, were recruited to reflect the clinical medical education system better. Furthermore, because one of our participants were the students, we chose people who were actively engaged in clinical practice and completed an internship (fifth, sixth and seventh year). Finally, we supplemented and deepened the data from web-based interviews with telephone interviews (video and voice call).

Our study included certain limitations in addition to these strengths. Although observation (as one of the important techniques of data collection in the situation analysis method) was not used in this study due to the critical conditions caused by the Covid-19 pandemic and the urgent need for social distancing, we attempted to overcome this limitation as much as possible by triangulation and the use of multiple data collection methods. We conducted this research at the undergraduate level of medical school, and the findings do not apply to postgraduate education. Finally, because teaching and learning in clinical education is context-dependent, transfer the findings to other countries should be done with caution.

### Implications for policy and practice

Our study has implications for educational policies and policy makers that can be considered in clinical education. The clinical learning environment is a complex and multi-layered social environment in which educational policymakers and medical and health professional educators should consider these numerous social layers, arenas, social worlds, and discourses while developing curricula and teaching.

Medical educators and clinical teachers should develop a shared language to bridge the gap between theory and practice. Hearing the patient’s voice in clinical education and involving them in teaching, curriculum development, evaluation, and the balance of power in practice to build interconnectedness between pedagogy and clinical care in clinical teaching are some of the additional consequences of this study.

Furthermore, other the implications of this research that policymakers should consider include: developing a more comprehensive curriculum based on the elements and components of situational maps, focusing on the context in which medical education happens, paying attention to individual and collective human elements and actors, non-human and technology elements, implicated and silent actors and actants, discursive constructions of individual and or collective human actors, discursive constructions of non-human actants, political and economic elements, sociocultural and symbolic elements, and temporal and spatial elements in clinical medical education.

In addition, Based on the present study results, while considering the horizontal axis of the positional map (components and elements to develop effective clinical teaching), the authors recommend other items to practice and support effective clinical teaching, achieve the goals and outcome of clinical education and preparation competent graduates. These include; monitoring the implementation of the curriculum to increase the alignment and conformity between the planned curriculum and the delivered curriculum and the curriculum experienced by students, developing symbiotic relationships at the macro-level, at the meso-level and at the micro-level, recruiting educational specialists (medical education specialists) to consult individual and collective, and optimizing clinical teachers’ educational efforts in medical schools, focusing on systematic training in clinical education, adapting and aligning the student’s job description to the learning objectives, developing the attitude of teachers and students towards clinical education as a career pathway, promoting and developing students’ autonomy in clinical learning.

### Implications for research practice and future researchers

To obtain data and more profound results in future research, it is preferable to use observation as one of the essential data collection approaches in situational analysis. Future researchers might employ situational analysis to better understand and enhance this methodology in medical education research by applying it to different concepts and situations (e.g., postgraduate medical education). Also, unlike the present study, which is based on situational analysis: grounded theory after the postmodern turn, future studies can use most popular form of interpretive analysis (situational analysis: grounded theory after the interpretive turn). Finally, other researchers in medical education can strengthen the discourse that “methodological development will lead to the excellence and development of medical education” by developing this method and newer ones.

## Conclusion

The situational analysis focuses on an important aspect of medical education: the context in which medical education occurs. This is exactly what keeps us alive and well in medical education, particularly clinical education. In this study, the teaching-learning situation in clinical training in undergraduate medical education in Iran based on post-structural ground theory was represented in three maps; situational, social worlds/arenas, and positional. The results of this research illustrate the complex teaching-learning environment and process in clinical medical education. Also, the findings of this research revealed key messages that were explained using Foucault’s discourse.

## Supplementary Information

**Additional file 1.**

## Data Availability

All data generated during this study are included in this published article (and its Supplementary information file). Also, the qualitative interviews questions used during the current study are available from the corresponding author on reasonable request.

## Footnotes

1 According to the rules of the Virtual School of Medical Education & Management of Shahid Beheshti University of Medical Sciences (SBUMS), two peer students (as opponent students) accompany another peer from the beginning of the proposal approval to the final defense of the dissertation. The purpose of this is to create interaction, mutual critical appraisal and development journey (especially in terms of research). Since this is a rule and defined task for all students, adding peer students as contributors to the manuscript is not mandatory and there is no problem in terms of research ethics.

## References

[1] Midgley K (2006). Pre-registration student nurses perception of the hospital-learning environment during clinical placements. Nurse Educ Today.

[2] Pollard C, Ellis L, Stringer E, Cockayne D (2007). Clinical education: a review of the literature. Nurse Educ Pract.

[3] Steinert Y, Basi M, Nugus P (2017). How physicians teach in the clinical setting: the embedded roles of teaching and clinical care. Med Teach.

[4] AlHaqwi AI, Taha WS (2015). Promoting excellence in teaching and learning in clinical education. J Taibah Univ Medical Sci.

[5] Leinster S (2009). Learning in the clinical environment. Med Teach..

[6] Ahmady S, Khani H (2022). The development of the framework of effective teaching-learning in clinical education: a Meta-synthesis approach. Educ Res Int.

[7] Merton RK, Reader GG, Kendall PC (1957). The student-physician. Introductory studies in the sociology of medical education.

[8] Spencer J (2003). Learning and teaching in the clinical environment. BMJ..

[9] Ramani S, Leinster S. AMEE Guide no. 34: teaching in the clinical environment. Med Teach 2008;30(4):347–364. 10.1080/01421590802061613 10.1080/01421590802061613 18569655

[10] Myers K, Covington K (2019). Analysis of the clinical education situation framework: a tool for identifying the root cause of student failure in the United States. J Educ Eval Health Prof.

[11] Kiguli S, Baingana R, Paina L, Mafigiri D, Groves S, Katende G, et al. Situational analysis of teaching and learning of medicine and nursing students at Makerere University College of health sciences. BMC Int Health Hum Rights 2011;11(1):1–9. 10.1186/1472-698X-11-S1-S3 10.1186/1472-698X-11-S1-S3 PMC3059475 21411003

[12] Hoffman KG, Donaldson JF (2004). Contextual tensions of the clinical environment and their influence on teaching and learning. Med Educ.

[13] Heydari A, Dadgar Moghaddam M, Garoui HE (2021). General medical education (extern and intern) in school of medicine. Mashhad University of Medical Sciences.

[14] Changiz T, Yamani N, Shaterjalali M (2019). The challenge of planning learning opportunities for clinical medicine: a triangulation study in Iran. BMC Med Educ..

[15] Clarke AE (2005). Situational analysis: grounded theory after the postmodern turn.

[16] Clarke AE, Friese C, Washburn R. Situational analysis in practice: mapping research with grounded theory. London: Routledge; 2016.

[17] Ministry of Health and Medical Education (MOHME) (2017). Curriculum of undergraduate medical education (general medicine curriculum) in Iran. Government of the Islamic Republic of Iran, Tehran.

[18] Ministry of Health and Medical Education (MOHME) (2020). National standards of general medicine. Government of the Islamic Republic of Iran, Tehran, Iran.

[19] Ministry of Health and Medical Education (MOHME) (2015). Fundamental standards of undergraduate medical education in medical schools and higher education centers of Iran. Government of the Islamic Republic of Iran, Tehran, Iran.

[20] Ministry of health and medical education (MOHME) (2015). Document of expected competencies of general medicine graduates. Government of the Islamic Republic of Iran, Tehran, Iran.

[21] Ministry of health and medical education (MOHME) (2015). Clinical education standards. Government of the Islamic Republic of Iran, Tehran, Iran.

[22] Ministry of Health and Medical Education (MOHME) (2009). Clinical education criteria of general medicine program. Government of the Islamic Republic of Iran, Tehran, Iran.

[23] Ministry of Health and Medical Education (MOHME) (2009). Executive regulations for dress code and students’ professional ethics in laboratory and clinical environments. Government of the Islamic Republic of Iran, Tehran, Iran.

[24] Ministry of Health and Medical Education (MOHME) (2009). Charter of patients’ rights in Iran. Government of the Islamic Republic of Iran, Tehran, Iran.

[25] Birks M, Mills J (2015). Grounded theory: a practical guide.

[26] Stern PN, Bryant A, Charmaz K (2007). On solid ground: essential properties for growing grounded theory. The Sage handbook of grounded theory.

[27] Koch T (1994). Establishing rigour in qualitative research: the decision trail. J Adv Nurs.

[28] Lincoln YS, Guba EG. Naturalistic inquiry. Newbury Park: Sage. 1985.

[29] Roberts C, Kumar K, Finn G (2020). Navigating the qualitative manuscript writing process: some tips for authors and reviewers. BMC Med Educ.

[30] Beigzadeh A, Yamani N, Sharifpoor E, Bahaadinbeigy K, Adibi P (2021). Teaching and learning in clinical rounds: a qualitative meta-analysis. J Emerg Pract Trauma.

[31] LaCombe MA (1997). On bedside teaching. Ann Intern Med.

[32] Foucault M. The birth of the clinic. London: Routledge; 2012.

