# Supplementary material for: The situational analysis of teaching-learning in clinical education in Iran: a postmodern grounded theory study
Source: BMC Med Educ. 2022 Jul 2;22:520. doi: 10.1186/s12909-022-03577-3 (PMC9250741; doi:10.1186/s12909-022-03577-3)
Supplement: Supplementary file 1 — Additional file 1. [file 12909_2022_3577_MOESM1_ESM.docx]

**Supplementary information file**

One of the interviewees says that in clinical education of general medicine in Iran, the roles of teaching and clinical care are inseparable. I think according to this person's point of view (medical education specialist), I should go to a student or clinical teacher and see how they think about this?

**Supplemental 1. An example of theoretical memos**

Reading one of the interviewees' answers, I realized that a clinical teacher considers what he or she does and needs to teach as components of the clinical education situation. But what this clinical teacher does, is not a mere reflection and picture of the situation of clinical education. According to this person's answer, I must go to students, medical education specialists and curriculum analysis. Of course, for now, I have to wait and see how other clinical teachers think. If other participants mention this, I need to ask more questions from future participants in telephone interviews.

**Supplemental 2. An example of methodological or operational memos**

**The first stage**

**The second stage**

**Basic sciences**

**Physiopathology/Semiology**

**Clerkship**

**The third stage**

**Internship**

**The fourth stage**

**Supplemental 3. An example of diagram memos**

**Supplemental 4. Relational analysis using the emerged situational map: focus on clinical education**

**Supplemental 5. Relational analysis using the emerged situational map: focus on teaching-learning in clinical education**

**Supplemental 6. Challenges and problems obstacle to effective teaching-learning in clinical education based on six positions**

**Position 1**

**Challenges and problems of curriculum**

- Lack of coordination between clinical education and community needs and a small amount of use of community-based medical education approach

- Discipline-Based /Subject-based Undergraduate Medical Curriculum

- Dominance of hospital-based clinical education

- Unclear definition and/ or disagreement on students' learning needs due to student non-engagement in the development of goals and other elements of the curriculum

- Different perceptions of the goals and outcomes of learning intended in clinical teaching

- The challenge of disagreement over sequencing in the curriculum and integration (there is a clear boundary between preclinical and clinical training)

- Planning limited authentic learning opportunities

- Inconsistency between the student's job description and learning objectives

- Lack of attention to patient involvement in clinical teaching (the patient is only a subject for teaching-learning)

- Symbiotic relationships at a very weak level between clinical teacher, student and patient in clinical education

- Low students’ readiness when entering the clinical environment

- Insufficient attention to students' autonomy in clinical learning

- Weakness in designing and implementing systematic assessment and timely feedback in clinical teaching

- Insufficient attention to assessment methods appropriate to clinical teaching

- Very limited use of problem-based learning (PBL) approach in clinical teaching

- Insufficient attention to the reflection on teaching-learning in clinical education

- Dominance of opportunistic strategies over systematic training program in clinical education

- Limited use of small groups teaching method in clinical teaching

**Position 2**

**Challenges and problems related to culture, behavior and attitude in clinical education**

- Lack of attitude towards clinical education as a career pathway

- Poor time management

- Resistance of students, educators and clinical teachers against innovative educational changes

- Preference of passive teaching methods and strategies over methods and strategies requiring active learning by students (student non-engagement)

- Lack of motivation in students and clinical teachers

- Limited opportunities to faculty development of medical schools (lack of culture of organizational support)

- Underestimating the role of teaching and the need to develop educational skills relative to the role of research and patient care by teachers and clinical educators

**Supplemental 6. Continued**

**Position 4**

**Challenges and problems related to environment, space and time in clinical education**

- Lack of adequate educational space (for example, disproportion number of students with the space and learning environment)

- Stressful and threatening educational environment and space/ context

- Undesirable educational climate

- Time limitations in clinical teaching

- Late clinical exposure in clinical education

- Unbalanced selection of clinical arenas in favor of subspecialty hospitals

**Position 5**

**Challenges and problems of financial and economic in clinical education**

- Insufficient budget (lack of financial resources in clinical education)

- Limited resources to purchase equipment, instructional materials and new educational technologies

**Position 6**

**Challenges and problems related to equipment and technology in clinical education**

- Poor educational aid tools and materials

- Lack of necessary infrastructure to use technology and new educational technologies in clinical education (such as; virtual reality [VR] augmented reality [AR], virtual patient [VP] or e-patient, etc.)

**Position 3**

**Challenges and problems of management and leadership in clinical education**

- Imbalance between students' learning needs and service delivery and patient care (Imbalance between the dual roles of educational/ teaching and clinical care)

- Inadequate and inefficient supervision on training in a clinical settings

- Poor administrative support due to high workload in clinical education

- Lack of mentoring in clinical learning environments

- Lack of effective management of learning resources by clinical educators

- Lack of competency-based pay system for clinical educators and teachers

- Lack of employment of educational specialists (medical education specialists) to consult individual and collective and optimize the educational efforts of teachers and clinical instructors in medical schools

- Lack of reward and incentive system for teaching in clinical education
